# Essential thrombocythemia treatment algorithm 2018

**DOI:** 10.1038/s41408-017-0041-8

**Published:** 2018-01-10

**Authors:** Ayalew Tefferi, Alessandro M. Vannucchi, Tiziano Barbui

**Affiliations:** 1 Division of Hematology, Department of Medicine, Mayo Clinic, Rochester, MN USA; 2 Department of Experimental and Clinical Medicine, CRIMM, Center Research and Innovation of Myeloproliferative Neoplasms, Azienda Ospedaliera Universitaria Careggi, University of Florence, Florence, Italy; 3 Research Foundation, Papa Giovanni XXIII Hospital, Bergamo, Italy

## Abstract

Current drug therapy for myeloproliferative neoplasms, including essential thrombocythemia (ET) and polycythemia vera (PV), is neither curative nor has it been shown to prolong survival. Fortunately, prognosis in ET and PV is relatively good, with median survivals in younger patients estimated at 33 and 24 years, respectively. Therefore, when it comes to treatment in ET or PV, less is more and one should avoid exposing patients to new drugs that have not been shown to be disease-modifying, and whose long-term consequences are suspect (e.g., ruxolitinib). Furthermore, the main indication for treatment in ET and PV is to prevent thrombosis and, in that regard, none of the newer drugs have been shown to be superior to the time-tested older drugs (e.g., hydroxyurea). We currently consider three major risk factors for thrombosis (history of thrombosis, *JAK2*/*MPL* mutations, and advanced age), in order to group ET patients into four risk categories: “very low risk” (absence of all three risk factors); “low risk” (presence of *JAK2*/*MPL* mutations); “intermediate-risk” (presence of advanced age); and “high-risk” (presence of thrombosis history or presence of both *JAK2*/*MPL* mutations and advanced age). Herein, we provide a point-of-care treatment algorithm that is risk-adapted and based on evidence and decades of experience.

## Introduction

The term myeloproliferative neoplasms (MPN) typically refers to essential thrombocythemia (ET), polycythemia vera (PV), and primary myelofibrosis (PMF)^1^; in addition, some patients with ET or PV might in time progress into a PMF-like post-ET or post-PV myelofibrosis^2^. As a group, ET, PV, and PMF share three mutually exclusive “driver” mutations, including *JAK2*, *CALR*, and *MPL*^3^. The most frequent driver mutation is *JAK2*V617F, found in ~99% of patients with PV, 55% ET, and 65% PMF^4^. The driver mutation distributions in ET and PMF are similar with 50–65% of the patients being *JAK2*V617F mutated, 15–30% being *CALR* mutated, and 4–8% being *MPL* mutated^4^, while 10–20% of the patients might not express any one of the three mutations (i.e., are triple-negative)^4^.

World Health Organization (WHO)-consistent diagnosis of ET requires a platelet count of ≥450 × 10(9)/L, presence of one of the three aforementioned driver mutations or in their absence the exclusion of other causes of thrombocytosis (reactive and clonal), and bone marrow morphologic assessment, especially for distinguishing ET from prefibrotic PMF and “masked” PV^5,6^. In addition to clonal thrombocytosis, a variable proportion of patients with ET might display mild splenomegaly, leukocytosis, microvascular symptoms, thrombotic and bleeding complications, increased occurrence of first trimester miscarriage, and time-dependent risk of leukemic transformation or fibrotic progression^7^.

Survival in patients with any one of the three *JAK2* mutation-enriched MPN is significantly shorter than that of the sex- and age-adjusted control population, with median estimates of 20 years for ET, 14 years for PV, and 6 years for PMF^8^. Causes of death include leukemic transformation, with 15-year estimates of ~2.1–5.3% for ET, 5.5–18.7% for PV, and more than 20% for PMF^9^. Fibrotic progression rates in ET and PV, during a similar time interval, are estimated at 4–11% and 6–14%, respectively^9^. To date, drug therapy has not been shown to modify the natural history of these diseases, prevent leukemic or fibrotic progression or prolong survival^10^. Current indication for drug therapy in ET and PV is to prevent thrombotic complications, especially in high-risk patients^7^. In the current review, we provide a risk-adapted treatment algorithm in ET that can be used in daily practice.

## Risk-adapted treatment algorithm in essential thrombocythemia

### Survival and its prognostic determinants

Life expectancy in ET is only mildly compromised with median survival for patients younger than 60 years of age approaching 33 years^8^. In addition to age, other clinical risk factors for survival in ET include leukocytosis and thrombosis history^11^. On the other hand, neither abnormal karyotype (detected in ~7% of patients)^12^ nor driver mutational status^13^ in ET has been shown to affect overall or leukemia-free survival; however, *JAK2/MPL*-mutated patients are significantly more thrombosis prone while *MPL*-mutated cases might be at a higher risk for fibrotic progression^13–15^.

A recent targeted sequencing study revealed that mutations or DNA variants, other than *JAK2*, *CALR*, or *MPL*, are found in ~53% of patients with ET with the most frequent being *TET2* (16%), *ASXL1* (11%), *DNMT3A* (6%), and *SF3B1* (5%)^16^. The particular study identified *SH2B3*, *SF3B1*, *U2AF1*, *TP53*, *IDH2*, and *EZH2* mutations as risk factors for overall, myelofibrosis-free or leukemia-free survival; at least one of these mutations was seen in ~15% of the patients and median survival of patients with and without adverse mutations were 9 and 22 years, respectively. Furthermore, the effect on survival from these adverse mutations was not accounted for by current clinically devised prognostic models and the observations were validated in an external cohort of patients^16^. Most recently, serum lactate dehydrogenase (LDH) level in ET was shown to correlate with shortened survival, suggesting its value as a biologically more accurate measure of myeloproliferation (as opposed to leukocytosis) and possible surrogate for occult prefibrotic PMF^17^.

Taking the above discussion into consideration, it is important to identify the risk factor-free subset of ET patients, since their survival might not be significantly different from the age- and sex-matched control population; such patients are represented by morphologic confirmation of WHO-defined ET (as opposed to prefibrotic PMF), younger age, absence of thrombosis history, absence of leukocytosis, normal LDH, and absence of *MPL* or other adverse mutations, as outlined above. On the other hand, the presence of risk factors for survival is currently not used to dictate treatment, since specific therapy in ET has not been shown to affect survival. Accordingly, although advised after securing insurance coverage and patient permission, we do not believe it is currently crucial to obtain next-generation sequencing (NGS) in ET. In other words, at the present time, identification of survival risk factors in ET is used to counsel patients and disease-monitoring purposes and not for treatment decisions.

### Thrombosis and its prognostic determinants

Current treatment in ET is primarily indicated for the purposes of preventing thrombotic complications, which might occur in 10–20% of patients. In this regard, the two-tiered traditional risk stratification considers two risk parameters: age >60 years and history of thrombosis. Accordingly, patients with either one of these two risk factors were classified as “traditional high-risk” and the absence of both risk factors defined the “traditional low-risk” groups. More recently, however, several studies have identified the presence of *JAK2*/*MPL* mutations as another independent risk factor for thrombosis in ET^18,19^. More specifically, risk factors for arterial thrombosis included thrombosis history, age >60 years, presence of *JAK2*V617F, leukocytosis, and CV risk factors and for venous thrombosis male gender^19^, while a lower risk of thrombosis was shown in patients with extreme thrombocytosis^19^ and in those with *CALR* mutations^20,21^.

### Contemporary risk stratification

Thrombosis data from 1019 patients with WHO-defined ET was recently re-analyzed^18^; among the “traditional low-risk” group, annual thrombosis rate was the lowest in patients who lacked both *JAK2*/*MPL* mutations and CV risk factors (0.44%), non-significantly higher in *JAK2*-unmutated patients with CV risk factors (1.05%) and significantly higher in *JAK2*-mutated patients with (2.57%) or without (1.59%) CV risk factors; there was no significant difference between *JAK2*-mutated “traditional low-risk” patients with or without CV risk factors. In the “traditional high-risk” group, the particular study^18^ identified thrombosis history as being significantly more detrimental than advanced age and also showed that the adverse effect of *JAK2* mutations was more apparent in patients whose high-risk status was determined by advanced age while its additional effect on patients with thrombosis history was limited; these observations from the revised international prognostic scoring system for essential thrombocythemia thrombosis were recently validated by another study^22^.

**Fig. 1:**
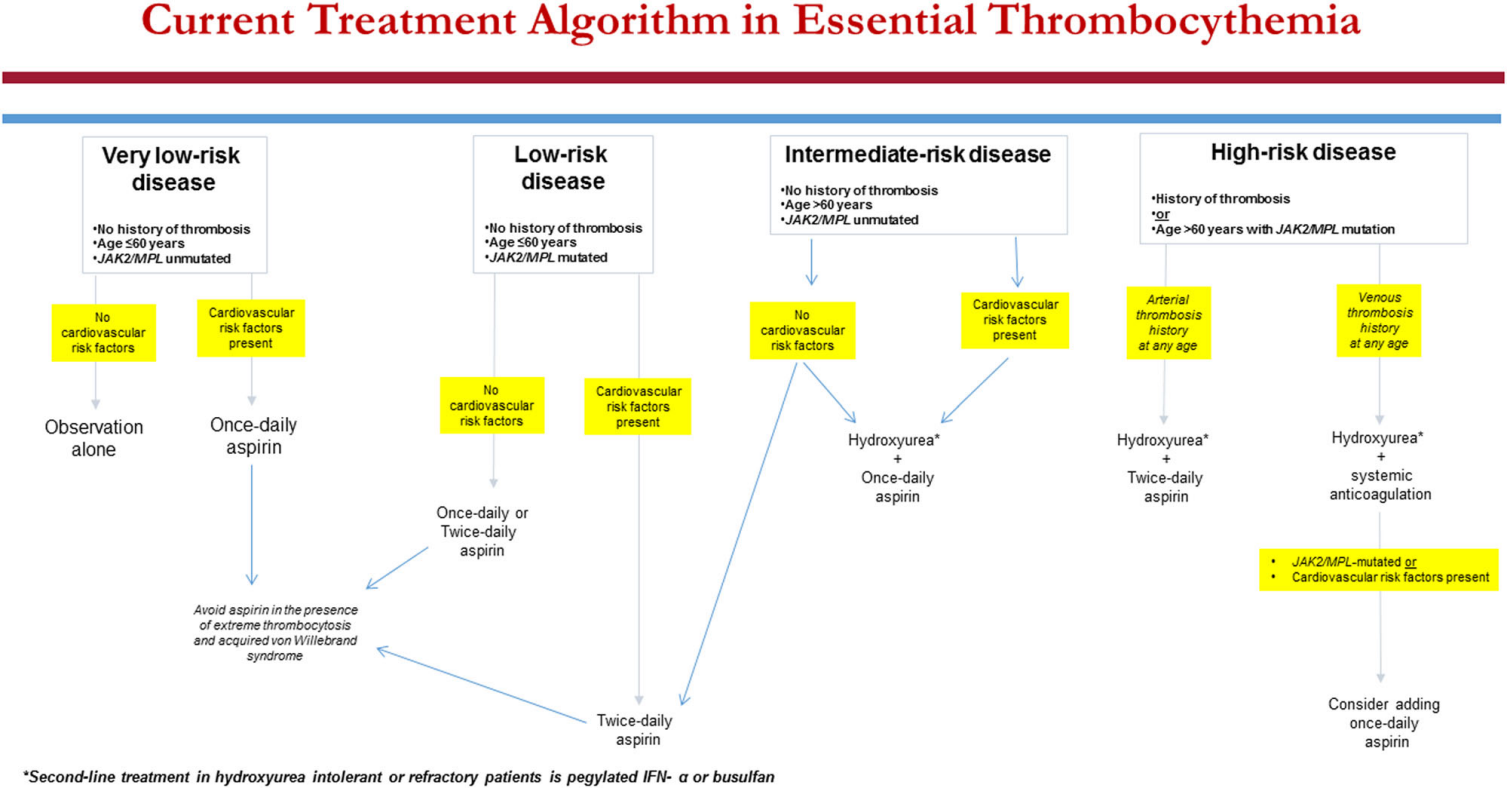
Current treatment algorithm in essential thrombocythemia Second-line treatment in hydroxyurea intolerant or refractory patients in pegylated IFN-α or busulfan

Based on the above, we currently consider four risk groups in ET: “very low-risk group” is defined by the absence of all three independent risk factors for thrombosis, including history of thrombosis, *JAK2*/*MPL* mutations, and advanced age; “low-risk” group is defined by the presence of *JAK2*/*MPL* mutations in otherwise younger patients without history of thrombosis; “intermediate-risk” group refers to *JAK2*/*MPL* unmutated older patients without thrombosis history; and “high-risk” group is defined by either presence of thrombosis history or presence of *JAK2*/*MPL* mutation in an older patient (Fig. 1).

### Risk-adapted therapy: “very low-risk” disease

At present, there is no evidence from prospective controlled studies to guide treatment recommendations for each one of the above-mentioned four risk categories in ET. Until such information becomes available, it is reasonable to simply observe patients with “very low-risk” disease without CV risk factors and consider once-daily aspirin therapy only in the presence of CV risk factors (Fig. 1). In other words, aspirin therapy in “very low-risk” ET should not be automatic, especially considering the fact that a substantial proportion of such patients display acquired von Willebrand syndrome (AVWS) with increased bleeding diathesis^23^; this particular complication is more likely to occur in the presence of extreme thrombocytosis^24^. Furthermore, other studies have suggested the value of aspirin therapy in reducing the risk of arterial thrombosis in low-risk ET associated with CV risk factors, but not otherwise^25^. Because patients with “very low-risk” ET are either *CALR* mutated or triple-negative, they usually display extreme thrombocytosis, which does not require specific treatment per se, regardless of how high the platelet count might be, as long as patient remains asymptomatic. On the other hand, if such patients with extreme thrombocytosis develop symptoms or bleeding complications, it is reasonable to use a cytoreductive agent, with a goal of keeping the platelet count commensurate with the resolution of the particular symptom.

### Risk-adapted therapy: “low-risk” disease

In “low-risk” ET (i.e., young *JAK2/MPL*-mutated without thrombosis history), the aforementioned recent studies have disclosed a residual risk of thrombosis despite management according to traditional treatment guidelines^18,26^. Therefore, it is reasonable to consider further optimization of aspirin therapy in such patients by following “twice-daily” rather than “once-daily” schedule, especially in the presence of CV risk factors^26^. The rationale for twice-daily aspirin dosing in “low-risk” *JAK2/MPL*-mutated ET patients is primarily based on emerging data on the inadequacy of once-daily aspirin dosing for 24-h optimal suppression of thromboxane-A2 synthesis, in the presence of high platelet turnover, and demonstration of superior biological efficacy in ET with twice-daily dosing^27,28^.

### Risk-adapted therapy: “intermediate-risk” disease

Recent studies have suggested that “advanced age,” by itself, was a weak risk factor for thrombosis and may not be as detrimental as thrombosis history^18,26^. These observations have led us to split the “traditionally high-risk” ET category into “intermediate risk,” defined by the presence of advanced age without history of thrombosis or *JAK2/MPL* mutations, and “high risk,” defined by presence of thrombosis history or presence of both advanced age and *JAK2/MPL* mutations. Such distinction is therapeutically relevant since it provides the option of avoiding cytoreductive therapy in *JAK2/MPL* unmutated older patients without history of thrombosis or CV risk factors (Fig. 1); in one of the aforementioned studies^18^, the annual risk of thrombosis in such patients was 1.44%, compared to 4.17% in the presence of both *JAK2* mutations and CV risk factors (*p* = 0.01), and was similar to that of “low-risk” patients (1.59–2.57%). Accordingly, we do not believe that it is mandatory to use cytoreductive therapy in such patients (Fig. 1).

### Risk-adapted therapy: “high-risk” disease

Decades ago, “high-risk” disease in ET was defined by the presence of one of three clinical parameters: history of thrombosis, advanced age, and long duration of thrombocytosis^29^. Subsequently, in a randomized study using hydroxyurea for high-risk disease, patients with platelet count >1500 × 10(9)/L were excluded because it was felt that such patients required treatment because of increased bleeding diathesis^30^. Over the years, it has become evident that extreme thrombocytosis in ET did not, by itself, increase thrombosis risk and might actually be associated with a reduced risk of arterial thrombosis^19,31^. Also, the bleeding diathesis associated with extreme thrombocytosis has been linked to AVWS^24^, which might occur both in the presence and absence of extreme thrombocytosis^23^, and is effectively screened for and managed appropriately. Therefore, platelet count per se should no longer be used for risk stratification in ET.

Regardless, management of “traditionally high-risk” ET has been primarily guided by results of a randomized study of hydroxyurea vs no cytoreductive treatment, in high-risk patients, with the goal of keeping the platelet count below 600 × 10(9)/L^30^; the study showed a statistically significant benefit for hydroxyurea therapy (thrombosis rate of 3.6 vs 24%). Since then, unsuccessful attempts have been made to improve upon hydroxyurea treatment in ET^32,33^. Accordingly, hydroxyurea, combined with once-daily aspirin therapy^34^, remains the standard of care for contemporarily classified “high-risk” patients (Fig. 1). However, there is room for improvement in our conventional treatment approach^18,26^ and we underscore the need to maximize anti-thrombotic activity, by shortening the aspirin dosing schedule to every 12 h, for patients with history of arterial thrombosis, and securing long-term systemic anticoagulation, in patients with history of venous thrombosis (Fig. 1). In addition, it is reasonable to continue with once-daily aspirin therapy, along with systemic anticoagulation, in patients who are at risk for arterial thrombosis (Fig. 1). In this regard, there is evidence for the additional value of aspirin therapy in the prevention of recurrent venous thrombosis^35,36^.

### Treatment options for hydroxyurea intolerant or refractory patients

There are currently four drugs to consider as second-line therapy in ET: pegylated interferon-α (IFN-α), busulfan, anagrelide, and pipobroman. Among these, our current choice for second-line therapy is pegylated IFN-α (starting dose 90 mcg SC weekly). Pegylated IFN-α treatment in ET has been shown to be relatively safe and effective, and has been associated with both clinical (70–80%) and molecular (10–20%) remissions in some patients, especially in the presence of *CALR* mutations^37,38^; however, the relevance of the latter observation, in terms of meaningful health outcome, remains uncertain. Busulfan (starting dose 2–4 mg/day) is a reasonable alternative drug for second-line therapy in ET and it too has been shown to be safe and effective as well as induce molecular remissions in both ET and PV^39,40^; in hydroxyurea intolerant or refractory patients with ET or PV, the drug was shown to induce durable hematologic response in the majority of patients and molecular response in a minority^41–43^. In addressing the ongoing concern regarding drug leukemogenicity, a large international study of over 1500 patients with PV found no evidence that implicated busulfan, IFN-α, or hydroxyurea, while confirming the particular association with pipobroman^44^. In a noteworthy vote of confidence regarding busulfan use in MPN, a prominent hematologist underscored the fact that busulfan displayed less DNA/RNA binding, compared to other alkylating agents, no inter- or intra-strand DNA binding and no immunosuppression^45^.

Anagrelide has been evaluated, in controlled studies, for its efficacy and safety as first-line therapy for ET^32,33^; the results of these studies suggested that anagrelide was not inferior to hydroxyurea in one study^33^, but might have been harmful to patients in the second study^32^. In the latter study, patients receiving anagrelide experienced higher incidences of arterial thrombosis, bleeding complications, and fibrotic progression. Similarly, non-controlled studies have suggested that more than a quarter of patients receiving anagrelide therapy become anemic while a lesser percentage experience renal insufficiency and cardiac complications including arrhythmia and cardiomyopathy^46–50^. Therefore, we currently consider anagrelide therapy only after failure of all other drug options, including hydroxyurea, IFN-α, and busulfan. Finally, despite some uncontrolled reports of safety and efficacy^51–53^, we currently do not recommend pipobroman treatment in ET, because of controlled evidence for leukemogenicity, seen in patients with PV^54^.

### Management during pregnancy

Current treatment recommendations in young women wishing to be pregnant or are pregnant include once-daily aspirin for “very low-risk” or “low-risk” disease and pegylated IFN-α for high-risk disease^55^. Both aspirin and IFN-α therapy have been shown to be safe for use during pregnancy and might be associated with lower miscarriage rates in women with ET^55–57^. The additional value of other measures, including platelet apheresis or low molecular weight heparin, is unclear and not recommended^58^.

## Conclusions

The most important first step in the management of ET is to confirm the accuracy of the diagnosis and make sure that other myeloid neoplasms, which might mimic ET in their presentation (e.g., prefibrotic PMF, masked polycythemia vera, chronic myeloid leukemia, refractory anemia with ring sideroblasts, and thrombocytosis), are excluded. Most patients with WHO-defined ET can expect a normal life expectancy with very low risk of leukemic transformation or fibrotic progression and a diagnosis of ET should not deter one from continuing with normal life activities, including sports, air travel, and pregnancy. Patients with ET should be informed about their driver mutational status and its prognostic and therapeutic implications. In this regard, aspirin therapy is very important for *JAK2*-mutated patients, because of their increased risk for arterial thrombosis. *MPL* mutations are infrequent in ET (~3%) and their presence raises the possibility of occult prefibrotic PMF or an increased risk of fibrotic progression. Observation alone remains a viable treatment option for “very low-risk” patients with ET while all other patients might benefit from aspirin therapy, in a once- or twice-daily schedule. In addition, cytoreductive treatment is strongly encouraged in patients with thrombosis history, and our first- and second-line drugs of choice in this regard are hydroxyurea and pegylated IFN-α, respectively. On the other hand, we no longer insist on the use of cytoreductive therapy in older patients without previous vascular events, provided they are *JAK2/MPL* unmutated.
